# Research and Remedies

**Published:** 1912-05-18

**Authors:** 


### RESEARCH AND REMEDIES.

### American Views on Epilepsy.

The treatment of the common form of epilepsy, which from ignorance of its true pathology and causation is usually termed idiopathic, is a subject upon which the last word has not yet been said. As a rule, the bromides serve the purpose better than any other drug; but drug treatment is only a part of the management of this serious disease, and in a certain proportion of cases even the bromides are useless. According to Weir-Mitchell, who inaugurated a discussion on this disease before the Philadelphia College of Physicians lately, this failure of bromide therapy is more common in minor epilepsy (*petit mal*) than in the convulsive cases; and he finds coal-tar products sometimes succeed when bromides thus fail. Not only are the latter sometimes useless for good; but occasionally they actually make the epileptic worse, not only in minor but (very rarely) also in major epilepsy. This author attaches but little importance to the exact base with which bromine is combined; but regards lithium bromide as possibly a trifle more effective than any of the others. He is in favour of increasing the dose grain by grain until the exact *minimum* is reached which will prevent the attacks. To avoid the skin eruptions he advises full doses of arsenic and hot baths; but a small percentage of patients, he finds, will get extensive rupial ulceration in spite of all precautions. Nitrate of silver, he believes, does good, but only when pushed so far as to cause argyria and a strong risk of permanent blackening of the skin. He has tried without success all kinds of dietary limitation; nor does he believe in forbidding coffee and tobacco. Amyl nitrite he still holds will check an impending fit, if inhaled when the *aura* first comes on: unfortunately so often there is no time to apply it.

Dercum endorses Weir-Mitchell's views upon diet. He insists especially on the value of open air, exercise, and attention to eliminatory system. He shares the opinions of German authors as to the value of withholding sodium chloride while bromides are being taken; and advises that sodium glycerophosphate be given along with the latter. The opium and the thyroid extract treatments he has found to do good in exceptional cases. The results of operation in idiopathic epilepsy have been, to judge by the surgical contributions to the discussion, as disappointing in America as elsewhere.

### The Ætiology of Cancer of the Stomach.

It is a very curious fact that wide differences of opinion prevail as to the association of stomachic cancer with previous ulcer. Knowledge of the symptoms, diagnosis, prognosis, and operative treatment of this disease has progressed in the past decade; but it is still unsettled whether those who have suffered from simple gastric ulcer are, or are not, more likely than healthy people to develop some day malignant disease in that organ. Weinstein, of New York, writing in the *Interstate Medical Journal*, emphasises his conviction that cancer of the stomach has a sudden, abrupt onset as a rule, and picks out those who have hitherto been in perfect health. The statistics from the Mayo clinic suggest the exact opposite, for they show that in 71 per cent. of cases the growth develops on the site of an ancient ulcer. On this side of the Atlantic just as great diversity of teaching exists, and two London consultants whom the writer met the other day over a gastric case expressed diametrically opposite views upon the subject. The matter is of importance from several standpoints. Thus an argument in favour of treating gastric ulcer by gastro-enterotomy is that thus the chance of subsequent malignant change is minimised; but this argument falls to the ground if, in fact, no such change is to be apprehended. Again, the history of hæmatemesis in past years has been held to point strongly to cancer, when the symptoms and signs of a patient have suggested such a possible diagnosis. But since an old ulcer may of itself, or by reason of adhesions, constitute a tumour palpable per abdomen, this history would be a point against malignancy, instead of for it, if Weinstein is right. This latter authority insists on the sudden onset of cancer of the stomach: the patient, he says, can date his symptoms to one day, or one meal, very often. Loss of appetite, loss of flesh and strength, and anæmia are progressive from the start; a gradual onset of stomach symptoms nearly always means a non-malignant disease. Physical signs he considers less important than the history.

